# The unspoken aspect of dementia care: sexuality

**DOI:** 10.1192/j.eurpsy.2025.2301

**Published:** 2025-08-26

**Authors:** M. M. Figueiredo, R. Pereira, V. Vila Nova

**Affiliations:** 1 Centro Hospitalar Setúbal, ULS Arrábida; 2 USF Santiago de Palmela, Setúbal, Portugal

## Abstract

**Introduction:**

Sexuality, although an essential component of human health, remains a controversial topic, shrouded in stigma, particularly in the context of dementia, where the expression of sexuality presents unique challenges.

**Objectives:**

The main objective of this work is to address the complexity of the biopsychosocial components of sexuality in patients with dementia, promoting a change in medical perspective and social attitudes.

**Methods:**

Evidence-based review, through research conducted on PubMed and selection of the most relevant studies on this topic, published in the last decade, using the keywords: “Sexuality” and “Dementia”.

**Results:**

Cognitive impairment can affect the frequency and satisfaction with sexual activity. Most studies focuses exclusively on the biological (and dysfunctional) component of sexuality, devaluing the challenges and barriers to the expression of this sexuality. The deterioration of cognitive processes, with emphasis on the involvement of the prefrontal cortex, can influence the ability to make decisions, setting boundaries and providing consent. Inappropriate sexual behaviors, such as disinhibition and hypersexuality, have an incidence of 7-25% in patients with dementia, and may require intervention psychopharmacological. These vulnerabilities result in an enormous challenge in terms of establishing a balance between autonomy and safety of these patients, sometimes resulting in neglect of sexual health in treatment environments.

**Conclusions:**

A comprehensive understanding of the sexuality of older adults with dementia is essential to improve the quality of life and clinical care of this population, highlighting the importance of accurate education and inclusive sexual orientation, creating safe spaces for dementia patients to explore and express their sexuality.

**Disclosure of Interest:**

None Declared

